# Supplementary material for: Postmortem Findings in Free-Ranging North American Beavers (Castor canadensis) Reveal Potential Threats to California’s Freshwater Ecosystems
Source: Animals (Basel). 2025 Jan 24;15(3):338. doi: 10.3390/ani15030338 (PMC11816171; doi:10.3390/ani15030338)

Supplementary material Table S1

Beaver	Case #	Sex	Age	Necropsy/Tissues	Manner of death	Location	Cause of death/primary disease	Clinical history	Pathological findings	Ancillary tests
1	160	Male	Juvenile	Necropsy	Natural death	Martinez, Contra Costa County	Baylisascariasis	Beaver was circling in the pond possibly for several days. On physical exam, the animal was blind, falling to the right, and extremely depressed. Azotemia, possible eosinophilia, and monocytosis.	Severe multifocal necrotizing and eosinophilic granulomatous encephalitis with intralesional neural larva migrans. Disseminated *Baylisascaris* sp. migration with granulomatous thymitis, pneumonia, myocarditis, hepatitis, glossitis, omentitis, enteritis, and diaphragmatic myositis	Molecular identification of *Baylisascaris* sp. (Brain): Negative. Virology: Rabies negative (Brain).
2	5185	Female	Juvenile (1y)	Necropsy	Natural death	Martinez, Contra Costa County	Baylisascaris sp.-suspected encephalitis	Found floating dead in creek. It is thought to be last year's kit (1-year-old). Frothy blood-tinged fluid at nares.	Moderate multifocal glial scars with mild nonsuppurative meningoencephalitis and plexus choroiditis. Pulmonary edema and congestion with intra-alveolar hemorrhages. Mild interstitial nephritis, hepatitis and myocarditis.	Molecular identification of *Baylisascaris* sp. (Brain): Negative. Immunohistochemistry: Toxoplasma gondii, Sarcocystis neurona, Canine Distemper Virus, West Nile virus, and Neospora caninum negative (Brain). Fecal flotation: No parasite eggs detected. Virology: Rabies negative (Brain).

										Bacteriology: *Salmonella* spp. negative (feces)
3	1280	Female	Juvenile (1y)	Necropsy	Natural death	Martinez, Contra Costa County	Baylisascaris sp.-suspected encephalitis	Found in Castro Street in the Alhambra Creek. Juvenile (last year's baby). No injuries noted. Unknown cause of death.	Moderate multifocal glial scars with moderate non-suppurative encephalitis. Hepatocellular swelling with intracytoplasmic eosinophilic vacuoles.	Molecular identification of *Baylisascaris* sp (Brain): Negative. Immunohistochemistry: *Toxoplasma gondii*, *Sarcocystis neurona*, Canine Distemper Virus, *West Nile* virus, and *Neospora caninum* negative (Brain). Fecal flotation: No parasite eggs detected. Bacteriology: *Salmonella* spp. negative (feces)
4	6352	Female	Adult (6y)	Necropsy	Euthanised	Martinez, Contra Costa County	Baylisascaris sp.-suspected encephalitis	Approximately 6-year-old multiparous female with a recently weaned litter. Observers have reported chronic eye problems that started in one eye and "have spread to both". Observers also report that other animals in the area have had problems with ocular larva migrans. On physical exam,	Multifocal glial scars. Eosinophilic and histiocytic interstitial pneumonia with vascular thombi. Malocclusion with secondary ulcerative proliferative gingivitis. Lymphocytic polyneuritis. Lymphocytic and neutrophilic conjuctivitis.	Molecular identification of Baylisascaris sp (Brain): Negative. Immunohistochemistry:Toxoplasma gondii, Sarcocystis neurona, Canine Distemper Virus, West Nile virus, and Neospora caninum negative (Brain). Fecal flotation: No parasite eggs detected. Virology: Rabies negative (Brain). Bacteriology: *Salmonella* spp. negative (feces)

								the upper incisor is broken with overgrowth of the lower incisor and penetration into the palate. The animal is in poor condition. Euthanized due to poor prognosis.		
5	14448	Male	Juvenile (1y)	Necropsy	Euthanised	Pittsburg, Contra Costa County	Baylisascariasis	Beaver presented to hospital after being found lethargic and easily approachable in a parking lot. Animal was obtunded and appeared non-visual. Radiographs were unremarkable. Anisocoria was present with the left eye very miotic, unable to look into the back of the eye. The lens of the right eye appeared very cloudy, as a potential cataract. A few	Severe multifocal eosinophilic granulomatous encephalitis with intralesional neural larva migrans, Disseminated *Baylisascaris* sp. migration with granulomatous lymphadenitis, myocarditis, enteritis and nephritis with visceral larva migrans.	Molecular identification of Baylisascaris sp (Brain): Negative. Virology: Rabies negative (Brain)

								superficial wounds on the tail, feet, over the back and a small abscess was present in the right ventral elbow. Patient showed no evidence of being visual. Beaver remained completely blind and showed no further improvements and it was euthanized.		
6	1	Fem ale	Adult	Necropsy	Natural death	Winters, Yolo County	Baylisascaris sp.-suspected encephalitis	Beaver was seen in vicinity where a dead beaver was found in public park that was not acting normal; was out during the day, approaching people; concern that this beaver may be diseased.	Moderate multifocal glial scars. Moderate multifocal nonsuppurative, eosinophilic encephalitis. Disseminated *Baylisascaris* sp. migration with granulomatous hepatitis, lymphadenitis, omentitis, and splenitis with visceral larva migrans. Cholangiocellular adenoma. Papillary renal adenoma.	Molecular identification of *Baylisascaris* sp (Brain): Negative. Aerobic culture: *Actinobacillus* sp and *Streptococcus* sp. (Liver). Immunohistochemistry: Canine Distemper virus negative (Brain); Pancytokeratin positive and vimentin negative (Liver and Kidney). Fecal flotation: No parasite eggs detected. Virology: Rabies negative (Brain). Bacteriology: *Salmonella* spp. negative (feces).

									Gastric nematodiasis. Cecal trematodiasis.	
7	2352	Fem ale	Adult	Necropsy	Euthani sed	Martine z, Contra Costa County	Baylisascaris sp.-suspected encephalitis	Beaver was found in the middle of the road, very easily caught up with a blanket. Patient displayed abnormal mentation with mild dried crusting around eyes and nares, thin body condition and minor signs of trauma around face and distal tip of tail. Blood work showed elevated WBC count. Patient continue to show signs of abnormal mentation, seemed non-visual, minimally responsive to surrounding and stimuli, not grooming self. After 24 days in rehab, the animal was on	Moderate multifocal glial scars and non-suppurative, eosinophilic encephalitis. Disseminated *Baylisascaris* sp. migration with granulomatous myocarditis, hepatitis, splenitis, gastritis, enteritis and colitis with visceral larva migrans. Moderate chronic interstitial pneumonia. Emaciation. Cecal trematodiasis.	Molecular identification of *Baylisascaris* sp (Brain): Negative. Aerobic culture: *Corynebacterium* sp., *E. coli,* and *Enterococcus casseliflavus* (Lung). Fecal flotation: No parasite eggs detected. Virology: Rabies negative (Brain)

								back in enclosure and appeared to have seizures. Euthansia was elected.		
8	1769 4	Fem ale	Adult	Necropsy	Natural death	Palo Alto, Santa Clara County	Baylisascariasi s	Found in Palo Alto, taken to rehabilitation center, died in care.	Severe multifocal granulomatous encephalitis with neural larva migrans. Severe multifocal glial scars. Severe diffuse chronic proliferative and lymphohistiocytic interstitial pneumonia with pneumocyte type II hyperplasia, fibrosis, smooth muscle hyperplasia, and rare plant-induced granulomas. Moderate multifocal fibrotic and papillary pleural hyperplasia. Mild multifocal lymphoplasmacytic tracheitis. Moderate multifocal fibrinonecrotizing colitis with mix-shaped bacteria	Aerobic culture: *Aeromonas hydrophila*, *Staphylococcus* sp., and *E. coli* (Lung); *Staphylococcus* sp., and *Corynebacterium inhibens* (Pleura); *Staphylococcus* sp. (Pericardium); and *E. coli* and mixed flora (Colon). Anaerobic culture: *Terrisporobacter* sp. (colon) Virology: Rabies negative (Brain). Bacteriology: Yersinia spp. negative (feces)

9	940	Fem ale	Juven ile	Necropsy	Natural death	Discover y Park, Sacrame nto County	Baylisascariasi s	Animal was found with most of the coat matted and dirty by mud. No use of right front limb and ataxia were observed.	Severe multifocal eosinophilic granulomatous encephalitis with intralesional neural larva migrans, axonal degeneration, and multifocal glial scars. Disseminated *Baylisascaris* sp. migration with granulomatous pneumonia, enteritis, myocarditis, and lymphonodular capsulitis with visceral larva migrans. Skin, subcutis, and latissimus dorsi muscle (right side of the thorax): Severe focally extensive chronic active necrotizing dermatitis, cellulitis, and polyphasic myositis with mix-shaped bacteria. Subcutis and muscles (right scapula and humerus): Severe multifocal chronic active necrosuppurative	Molecular identification of *Baylisascaris* sp (Brain): Positive (*B. procyonis* 99.91% of identity). Aerobic and anaerobic culture: *E.coli*, mixed flora, mixed coliforms, and *Bacteroides thetaiotaomicron* (Colon). Fecal flotation: No parasite eggs detected. Virology: Rabies negative (Brain). Bacteriology: Yersinia spp. and Salmonella spp. negative (feces)

									cellulitis and myositis with mix-shaped bacteria. Severe diffuse acute superficial necrotizing colitis with mix-shaped bacteria.	
10	D106 59	Male	Adult	Necropsy	Natural death	Lake Tahoe, El Dorado County	Tularemia	Beaver was found dead at Lake Baron in Tahoe Paradise Park, Lake Tahoe, El Dorado, CA, and submitted to Lake Tahoe Wildlife Care. This is the second beaver found dead in this park. Per the district biologist, there are depredation permits along the So. Upper Truckee Road area; however, the area where this beaver was found is too far a distance to be the same beaver.	Severe acute random necrotizing serositis, hepatitis, interstitial pneumonia, splenitis, orchitis, myocarditis, choroiditis and enteritis. Gastric nematodiasis.	Special aerobic culture (California Department of Public Health): *Francisella tularensis*
11	4395	Male	Adult	Necropsy	Natural death	Napa Valley, Napa County	*Listeria monocytogen es* encephalitis	Swimming in circles in fountain all day. Animal might be walking with a	Severe necrotizing and histiocytic meningoencephaliti s, ependymitis, and choroiditis with	Bacterial Culture in cold enrichment: *Listeria monocytogenes* (Brainstem). Immunohistochemistry:

								slight ataxia, but her ambulation is lumbered. Proptosis of the lef eye without reaction to light. After six days of being found, beaver was found rolling and seizing, and died before any action was taken.	gram-positive rods. Multifocal glial scars. Cerebral *Toxoplasma gondii* cysts. Ulcerative keratitis, conjunctivitis with mix-shaped bacteria and yeasts. Severe pleocellular colitis and crypt necrosis. Mild focal granulomatous typhlitis with visceral larva migrans.	*Listeria monocytogenes* and *Toxoplasma gondii* positive (Brain); *Sarcocystis neurona*, *Canine Distemper Virus* and *Neospora caninum* negative (Brain). Fecal flotation: No parasite eggs detected. Molecular identification and genotypification of *Toxoplasma gondii*: Type X variant (Brain). Virology: Rabies negative (Brain).
12	16464	Female	Juvenile	Necropsy	Euthanised	Tanzanite Park, Sacramento County	*Staphylococcus aureus* bronchopneumonia	A juvenile beaver presented for care in response to a diesel spill in the animal's pond. On initial exam, the animal was noted to be under-conditioned at BCS 2/5 with a greasy coat and burns on the paws. It was transferred to a wildlife rehabilitation rescue after routine washing. While in the center, the animal was	Severe multifocal to coalescing subacute necropsuppurative bronchopneumonia and fibrinosuppurative pleuritis with gram-positive bacterial colonies	Aerobic culture: *Staphylococcus aureus* (Lung, Liver, Pleura, and Peritoneum). Anaerobic culture: No anaerobes detected (Lung). Fecal flotation: No parasite eggs detected. Virology: Rabies negative (Brain). Bacteriology: *Yersinia* spp. and *Salmonella* spp. negative (feces)

								noted as active, eating well, water-proofing appeared adequate, and having normal stools. Beaver was observed to acutely hide away and became severely lethargic. Hyperthermia was noted and euthanasia was elected		
13	5802	Male	Adult	Necropsy	Natural death	Pismo State beach, San Luis Obispo County	Bacterial bronchopneu monia	Found death without any previous clinical signs.	Severe multifocal to coalescing suppurative bronchopneumonia with gram-negative rods, gram-positive cocci, and rare foreign material. Minimal multifocal lymphoplasmacytic tracheitis. Moderate multifocal granulomatous lymphadenitis with mineralization and bacteria. Cecal trematodiasis	Aerobic culture: *Rahnella aquatilis*, *Streptococcus uberis*, and *Hafnia alvei* (Lung).Anaerobic culture: No anaerobes detected (Lung). Fecal flotation: No parasite eggs detected. Virology: Rabies negative (Brain). Bacteriology: *Yersinia* spp. and *Salmonella* spp. negative (feces)
14	351	Male	Adult	Necropsy	Natural death	Napa Valley, Napa County	Bacterial encephalitis	Hyperthermic and in respiratory distress once admitted and	Moderate multifocal suppurative meningoencephaliti s and	Aerobic culture: *Acinetobacter towneri* (Brain). Culture in cold enrichment: No Listeria detected (Brain). Fecal

								died overnight. Prior to coming into rehab, Beaver was reported to have been swimming in circles in the river.	fibrinosuppurative choroiditis with gram negative coccobacilli. Focal glial scar and mineralization. Moderate multifocal neutrophilic and histiocytic pneumonia with foreign material (plant) and bacteria. Mild focal granulomatous colitis with visceral larva migrans	flotation: *Giardia* spp. cysts >10 per slide. Immunohistochemistry: *Listeria monocytogenes* negative (Brain). Virology: Rabies negative (Brain). Bacteriology: *Yersinia* spp. and *Salmonella* spp. negative (feces)
15	3126	Male	Adult	Necropsy	Natural death	Oakley, Contra Costa County	Bacterial myofascitis	Beaver was found by East Bay Regional Park District staff at Big Break in a slough. Animal had shallow breathing and its legs were twitching. It died moments after it was found.	Skeletal muscle, fascia, salivary gland, and adipose tissue (Pectoral region): Severe diffuse chronic-active fibrinonecrotizing myofascitis, cellulitis, and sialoadenitis with fibrinoid vasculitis, thrombi and gram-negative bacterial colonies. Severe diffuse edema with thrombi and embolic interstitial pneumonia and fibrinous pleuritis. Heart (right atrium):	Aerobic culture: Aeromonas bestiarum (Pectoral and cervical muscles, lung, and liver), and Pasteurella multocida (Pectoral muscles). Anaerobic culture: No anaerobes detected (Pectoral and cervical muscles). No parasite eggs detected. Virology: Rabies negative (Brain). Bacteriology: *Yersinia* spp. negative (feces)

									Thrombus. Cecal trematodiasis.	
16	2416	Fem ale	Adult	Tissues	Euthani sed	Sacrame nto County	Squamous cell carcinoma	Killed (Depredation) Sacramento County. Trapped by Wildlife Service. A skin mass of 8.5x7x1.5cm, firm, hairless, red-brown, nodular, and pedunculated is located in the right flank. The mass is internally white-pale tan and a few 0.5x0.5cm multifocal grainy areas.	Skin: Squamous cell carcinoma. Severe diffuse acute neutrophilic endometritis with bacteria. Castor gland: Severe diffuse neutrophilic and lymphoplasmacytic adenitis with squamous metaplasia.	Immunohistochemistry: Pancytokeratin positive and vimentin negative (skin mass). Virology: Rabies negative (Brain)
17	4782	Male	Adult	Necropsy	Euthani sed	Courtlan d, Sacrame nto County	Trauma	Beaver was brought to Sac Wildlife Care rehab facility because possible injury and was euthanized. Postmortem radiograph indicated a fracture of the pubic bone, possible trauma.	Multiple and complete pelvic fractures, myofascial hemorrhages, and edema. Subcutaneous hemorrhages and edema in head and inguinal region. Mild granulomatous hepatitis with mineralization and Capillaria sp.	Fecal flotation: Coccidia oocysts and Strongylid eggs. Molecular identification and sequencing of *Capillaria* (*Calodium*) sp. (Liver). Virology: Rabies negative (Brain)

									(Synonym: Calodium sp.). Sarcocycstis sp. in tongue. Gastric nematodiasis.	
18	1449 4	Male	Adult	Necropsy	Natural death	Sutter County	Capture cardiomyopathy	Beaver was captured and processed. After two days, animal showed ataxia, hypersalivation and noisy respiratory effort.	The pericardial sac contains fibrin and hemorrhage. Moderate multifocal monophasic myocardial necrosis and degeneration. Moderate multifocal glial "track" scars with mild adjacent non-suppurative encephalitis. Mild multifocal granulocytic and histiocytic pneumonia	Aerobic culture: *Corynebacterium* sp (Lung, Liver, and Pericardium). Fecal flotation: Coccidia oocysts >10 per slide and *Giardia* spp. cysts >10 per slides. Virology: Rabies negative (Brain). Bacteriology: Salmonella spp. negative (feces)

supplementary material
Table S2

**Antibody specificity**	**Source**	**Host/Isotype**	**Dilution Primary antibody**	**Incubation time and temperature**	**Positive control**
Pancytokeratin	Milipore Sigma	Mouse / IgG1	1000	45min / RT	Skin of beaver
Vimentin	Milipore Sigma	Mouse / IgG1	1000	45min / RT	Skin of beaver
Francisella tularensis	Thermo Scientific	Mouse / IgG3	2000	30min / RT	Liver of rabbit
Listeria monocytogenes	DIFCO	Rabbit/polyclonal antiserum	1500	30min / RT	Brain of goat
Toxoplasma gondii	DAKO	Rabbit/polyclonal antiserum	1500	1h / RT	Brain of raccoon
Sarcocystis neurona	Non commercial	Rabbit/polyclonal antiserum	1600	30min / RT	Brain of horse
Neospora caninum	Non commercial	Rabbit/polyclonal antiserum	400	1h / RT	Brain of bovine fetus
Canine Distemper virus	VMRD	Mouse/IgG2b	4500	30min / 4°C	Brain of raccoon
West Nile virus	ATCC	Mouse/hyperimmune ascitic fluid	200	30min / RT	Heart of red-tailed hawk

supplementary material Table S3

Description	Scientific Name	Max Score	Total Score	Query Cover	E value	Per. ident	Acc. Len	Accession
Baylisascaris procyonis isolate X7425 large subunit ribosomal RNA gene, partial sequence	Baylisascaris procyonis	1967	1967	98%	0	99.91	1067	MG937775.1
Baylisascaris procyonis isolate X7523 large subunit ribosomal RNA gene, partial sequence	Baylisascaris procyonis	1967	1967	98%	0	99.91	1067	MG937774.1
Baylisascaris columnaris isolate X8554 large subunit ribosomal RNA gene, partial sequence	Baylisascaris columnaris	1960	1960	98%	0	99.81	1067	MG937772.1
Baylisascaris columnaris isolate X8381 large subunit ribosomal RNA gene, partial sequence	Baylisascaris columnaris	1960	1960	98%	0	99.81	1067	MG937773.1
Baylisascaris devosi isolate X8498 large subunit ribosomal RNA gene, partial sequence	Baylisascaris devosi	1932	1932	98%	0	99.34	1067	MG937776.1
Baylisascaris transfuga isolate X8321 large subunit ribosomal RNA gene, partial sequence	Baylisascaris transfuga	1871	1871	98%	0	98.31	1067	MG937779.1
Baylisascaris transfuga isolate X8321 28S ribosomal RNA gene, partial sequence	Baylisascaris transfuga	1871	1871	98%	0	98.31	1067	MH551546.1
Baylisascaris ailuri isolate X8548 large subunit ribosomal RNA gene, partial sequence	Baylisascaris ailuri	1868	1868	98%	0	98.31	1067	MG937778.1
Baylisascaris transfuga isolate X8743 large subunit ribosomal RNA gene, partial sequence	Baylisascaris transfuga	1866	1866	98%	0	98.22	1067	MG937780.1
Baylisascaris schroederi isolate X8549 large subunit ribosomal RNA gene, partial sequence	Baylisascaris schroederi	1860	1860	98%	0	98.13	1067	MG937777.1
Baylisascaris procyonis isolate N210 large subunit ribosomal RNA gene, partial sequence	Baylisascaris procyonis	1851	1851	92%	0	99.9	1005	AY821774.1
Baylisascaris tasmaniensis isolate X8505 large subunit ribosomal RNA gene, partial sequence	Baylisascaris tasmaniensis	1799	1799	98%	0	97.09	1067	MG937781.1
Baylisascaris devosi isolate Iran-Ramsar large subunit ribosomal RNA gene, partial sequence	Baylisascaris devosi	1797	1797	91%	0	99.2	998	MN960313.1
Baylisascaris devosi isolate Kamchatka_TK3 large subunit ribosomal RNA gene, partial sequence	Baylisascaris devosi	1790	1790	91%	0	99.29	990	KY465564.1

Ascaris lumbricoides 5.8S ribosomal RNA and 28S ribosomal RNA genes, partial sequence	Ascaris lumbricoides	1783	1783	100%	0	96.31	3979	AY210806.1
Ascaris suum 28S large subunit ribosomal RNA gene, partial sequence	Ascaris suum	1772	1772	100%	0	96.13	1146	FJ418792.1
Toxascaris leonina isolate X8696 large subunit ribosomal RNA gene, partial sequence	Toxascaris leonina	1755	1755	98%	0	96.34	1067	MG937784.1
Ascaris suum isolate X8719 large subunit ribosomal RNA gene, partial sequence	Ascaris suum	1749	1749	98%	0	96.25	1067	MG937782.1
Ascaris suum isolate N7 large subunit ribosomal RNA gene, partial sequence	Ascaris suum	1687	1687	94%	0	96.21	1030	AY821773.1
Baylisascaris laevis isolate UAM130585 large subunit ribosomal RNA gene, partial sequence	Baylisascaris laevis	1652	1652	86%	0	98.31	943	ON994377.1
Baylisascaris laevis isolate MRFB1 large subunit ribosomal RNA gene, partial sequence	Baylisascaris laevis	1633	1633	85%	0	98.39	930	ON994376.1
Baylisascaris laevis isolate MSBP24594 large subunit ribosomal RNA gene, partial sequence	Baylisascaris laevis	1626	1626	85%	0	98.28	929	ON994378.1
Parascaris equorum isolate X8740 large subunit ribosomal RNA gene, partial sequence	Parascaris equorum	1524	1524	98%	0	92.5	1069	MG937783.1
Parascaris equorum isolate N8 large subunit ribosomal RNA gene, partial sequence	Parascaris equorum	1500	1500	97%	0	92.34	1057	AY821775.1
Baylisascaris procyonis isolate HX1 large subunit ribosomal RNA gene, partial sequence	Baylisascaris procyonis	1382	1382	68%	0	100	748	OR457646.1
Baylisascaris procyonis 28S ribosomal RNA gene, partial sequence	Baylisascaris procyonis	1367	1367	68%	0	99.87	743	U94753.1
Toxocara cati isolate 1079 large subunit ribosomal RNA gene, partial sequence	Toxocara cati	1362	1362	99%	0	89.48	1098	OR728768.1
Hysterothylacium reliquens 28S ribosomal RNA gene, partial sequence	Hysterothylacium reliquens	1341	1341	99%	0	89.29	1090	MK558800.1
Baylisascaris procyonis isolate Bp9 28S ribosomal RNA gene, partial sequence	Baylisascaris procyonis	1327	1327	66%	0	100	718	KC543470.1

Raphidascaris acus isolate N182 large subunit ribosomal RNA gene, partial sequence	Raphidascaris acus	1325	1325	96%	0	89.38	1052	AY821772.1
Anisakis physeteris small subunit ribosomal RNA gene, partial sequence; internal transcribed spacer 1, 5.8S ribosomal RNA gene, and internal transcribed spacer 2, complete sequence; and large subunit ribosomal RNA gene, partial sequence	Skrjabinisakis physeteris	1323	1323	100%	0	88.72	2141	ON622794.1
Baylisascaris columnaris isolate K19 28S ribosomal RNA gene, partial sequence	Baylisascaris columnaris	1321	1321	66%	0	99.86	718	KC543469.1
Baylisascaris columnaris isolate K15 28S ribosomal RNA gene, partial sequence	Baylisascaris columnaris	1319	1319	66%	0	99.86	717	KC543467.1
Baylisascaris columnaris isolate K16 28S ribosomal RNA gene, partial sequence	Baylisascaris columnaris	1315	1315	66%	0	99.72	718	KC543466.1
Toxocara vitulorum 28S large subunit ribosomal RNA gene, partial sequence	Toxocara vitulorum	1312	1312	100%	0	88.49	1147	FJ418790.1
Pseudoterranova decipiens isolate PTN1 18S ribosomal RNA gene, partial sequence; internal transcribed spacer 1, 5.8S ribosomal RNA gene, and internal transcribed spacer 2, complete sequence; and 28S ribosomal RNA gene, partial sequence	Pseudoterranova decipiens	1308	1308	100%	0	88.47	2197	JQ673262.1
Pseudoterranova decipiens isolate PTN2 18S ribosomal RNA gene, partial sequence; internal transcribed spacer 1, 5.8S ribosomal RNA gene, and internal transcribed spacer 2, complete sequence; and 28S ribosomal RNA gene, partial sequence	Pseudoterranova decipiens	1308	1308	100%	0	88.47	2197	JQ673263.1
Baylisascaris procyonis genotype 3 28S ribosomal RNA gene, partial sequence	Baylisascaris procyonis	1299	1299	64%	0	99.86	705	KP843605.1

Skrjabinisakis physeteris isolate MS-ES-2015 18S ribosomal RNA gene, partial sequence; internal transcribed spacer 1, 5.8S ribosomal RNA gene, and internal transcribed spacer 2, complete sequence; and 28S ribosomal RNA gene, partial sequence	Skrjabinisakis physeteris	1297	1297	99%	0	88.45	1942	KY826440.1
Baylisascaris procyonis genotype 2 28S ribosomal RNA gene, partial sequence	Baylisascaris procyonis	1295	1295	64%	0	99.72	705	KP843604.1
Baylisascaris schroederi isolate Am1 28S ribosomal RNA gene, partial sequence	Baylisascaris schroederi	1286	1286	68%	0	97.98	743	JN257013.1
Baylisascaris transfuga 28S ribosomal RNA gene, partial sequence	Baylisascaris transfuga	1284	1284	68%	0	97.85	743	U94754.1
Contracaecum ovale isolate Gre3nem1 large subunit ribosomal RNA gene, partial sequence	Contracaecum ovale	1280	1280	97%	0	88.48	1368	OL470528.1
Baylisascaris ailuri isolate Af1 28S ribosomal RNA gene, partial sequence	Baylisascaris ailuri	1279	1279	68%	0	97.71	743	JN257012.1
Baylisascaris transfuga isolate Uap1 28S ribosomal RNA gene, partial sequence	Baylisascaris transfuga	1279	1279	68%	0	97.71	743	JN257009.1
Anisakis typica 18S rRNA gene (partial), ITS1, 5.8S rRNA gene, ITS2 and 28S rRNA gene (partial)	Anisakis typica	1277	1277	100%	0	87.96	2422	HF911524.1
Pseudoterranova decipiens isolate N167 large subunit ribosomal RNA gene, partial sequence	Pseudoterranova decipiens	1275	1275	98%	0	88.28	1069	AY821763.1
Baylisascaris transfuga isolate Um1 28S ribosomal RNA gene, partial sequence	Baylisascaris transfuga	1273	1273	68%	0	97.58	743	JN257008.1
Anisakis sp. SAN-2004 isolate N243 large subunit ribosomal RNA gene, partial sequence	Anisakis sp. SAN-2004	1271	1271	98%	0	88.18	1069	AY821759.1
Contracaecum sp. ALS-2019 large subunit ribosomal RNA gene, partial sequence	Contracaecum sp. ALS-2019	1266	1266	95%	0	88.81	1033	MN526256.1
Pseudoterranova decipiens isolate N138 large subunit ribosomal RNA gene, partial sequence	Pseudoterranova decipiens	1251	1251	97%	0	88.03	1063	AY821761.1
Baylisascaris potosis gene for 28S ribosomar RNA, partial sequence	Baylisascaris potosis	1247	1247	64%	0	98.72	702	AB893608.1

Anisakis simplex 'C' SAN-2004 isolate N134 large subunit ribosomal RNA gene, partial sequence	Anisakis berlandi	1245	1245	97%	0	88.03	1056	AY821754.1
Hysterothylacium fortalezae 28S ribosomal RNA gene, partial sequence	Hysterothylacium fortalezae	1234	1234	100%	0	87.29	1104	MK558799.1
Baylisascaris transfuga isolate Bt1 28S ribosomal RNA gene, partial sequence	Baylisascaris transfuga	1232	1232	66%	0	97.63	718	KC543471.1
Skrjabinisakis brevispiculata isolate GOM1 28S ribosomal RNA gene, partial sequence	Skrjabinisakis brevispiculata	1232	1232	95%	0	88.25	1043	KX098560.1
Hysterothylacium reliquens large subunit ribosomal RNA gene, partial sequence	Hysterothylacium reliquens	1230	1230	98%	0	87.69	1089	MZ320999.1
Baylisascaris transfuga 28S ribosomal RNA gene, partial sequence	Baylisascaris transfuga	1223	1223	65%	0	97.62	713	MW026407.1
Pseudoterranova decipiens isolate N166 large subunit ribosomal RNA gene, partial sequence	Pseudoterranova decipiens	1221	1221	95%	0	87.97	1041	AY821762.1
Contracaecum sp. SAN-2004 isolate N133 large subunit ribosomal RNA gene, partial sequence	Contracaecum sp. SAN-2004	1214	1214	94%	0	88.02	1029	AY821769.1
Contracaecum radiatum large subunit ribosomal RNA gene, partial sequence	Contracaecum radiatum	1214	1214	90%	0	89.04	980	AF226577.1
Pseudoterranova decipiens isolate N137 large subunit ribosomal RNA gene, partial sequence	Pseudoterranova decipiens	1212	1212	95%	0	87.9	1036	AY821760.1
Ascaris lumbricoides 28S ribosomal RNA gene, partial sequence	Ascaris lumbricoides	1206	1206	68%	0	95.96	743	U94751.1
Contracaecum miroungae large subunit ribosomal RNA gene, partial sequence	Contracaecum mirounga	1203	1203	90%	0	88.83	980	AF226581.1
Ascaris suum 28S ribosomal RNA gene, partial sequence	Ascaris suum	1201	1201	68%	0	95.83	743	U94752.1
Contracaecum sp. SAN-2004 isolate N164 large subunit ribosomal RNA gene, partial sequence	Contracaecum sp. SAN-2004	1199	1199	95%	0	87.74	1029	AY821770.1
Toxascaris leonina isolate PTa1 28S ribosomal RNA gene, partial sequence	Toxascaris leonina	1190	1190	68%	0	95.56	743	JN256999.1
Toxascaris leonina isolate CI1 28S ribosomal RNA gene, partial sequence	Toxascaris leonina	1190	1190	68%	0	95.56	743	JN257002.1

Toxascaris leonina 28S ribosomal RNA gene, partial sequence	Toxascaris leonina	1190	1190	68%	0	95.56	743	U94769.1
Hysterothylacium pelagicum large subunit ribosomal RNA gene, partial sequence	Hysterothylacium pelagicum	1188	1188	90%	0	88.55	982	AF226590.1
Contracaecum osculatum strain B large subunit ribosomal RNA gene, partial sequence	Contracaecum osculatum	1182	1182	90%	0	88.43	981	AF226580.1
Anisakis typica isolate 06556 28S ribosomal RNA gene, partial sequence	Anisakis typica	1182	1182	95%	0	87.37	1047	KX098562.1
Contracaecum osculatum large subunit ribosomal RNA gene, partial sequence	Contracaecum osculatum	1182	1182	90%	0	88.43	981	AF226576.1
Toxascaris leonina isolate W-PTt1 28S ribosomal RNA gene, partial sequence	Toxascaris leonina	1182	1182	68%	0	95.42	742	JN256998.1
Phocascaris sp. 112000 large subunit ribosomal RNA gene, partial sequence	Phocascaris sp. 112000	1177	1177	90%	0	88.32	981	AF226575.1
Hysterothylacium reliquens 28S ribosomal RNA gene, partial sequence	Hysterothylacium reliquens	1173	1173	91%	0	88.1	991	KU527061.1
Contracaecum micropapillatum large subunit ribosomal RNA gene, partial sequence	Contracaecum micropapillatum	1171	1171	90%	0	88.28	982	AF226587.1
Contracaecum spiculigerum gene for 28S ribosomal RNA, partial sequence	Contracaecum spiculigerum	1168	1168	63%	0	97.24	1658	AB189984.1
Contracaecum osculatum baicalensis large subunit ribosomal RNA gene, partial sequence	Contracaecum osculatum baicalensis	1162	1162	90%	0	88.02	981	AF226589.1
Raphidascaris gigi gene for large subunit ribosomal RNA, partial sequence, isolate: WAKA-3	Raphidascaris gigi	1162	1162	95%	0	86.99	1033	AB558480.1
Contracaecum osculatum strain A large subunit ribosomal RNA gene, partial sequence	Contracaecum osculatum	1160	1160	90%	0	88.02	981	AF226583.1
Contracaecum sp. SAN-2004 isolate N168 large subunit ribosomal RNA gene, partial sequence	Contracaecum sp. SAN-2004	1155	1155	91%	0	87.7	994	AY821768.1
Contracaecum septentrionale large subunit ribosomal RNA gene, partial sequence	Contracaecum septentrionale	1149	1149	90%	0	87.83	982	AF226588.1

Baylisascaris transfuga 28S ribosomal RNA gene, partial sequence	Baylisascaris transfuga	1149	1149	62%	0	97.47	676	MF419819.1
Contracaecum eudyptulae large subunit ribosomal RNA gene, partial sequence	Contracaecum eudyptulae	1144	1144	90%	0	87.73	982	AF226586.1
Contracaecum rudolphii strain A large subunit ribosomal RNA gene, partial sequence	Contracaecum rudolphii	1144	1144	90%	0	87.73	982	AF226585.1
Contracaecum microcephalum large subunit ribosomal RNA gene, partial sequence	Contracaecum microcephalum	1138	1138	90%	0	87.63	981	AF226573.1
Contracaecum ogmorhini large subunit ribosomal RNA gene, partial sequence	Contracaecum ogmorhini	1133	1133	90%	0	87.53	982	AF226582.1
Contracaecum rudolphii strain B large subunit ribosomal RNA gene, partial sequence	Contracaecum rudolphii	1133	1133	90%	0	87.53	982	OP912388.1
Hysterothylacium auctum large subunit ribosomal RNA gene, partial sequence	Hysterothylacium auctum	1133	1133	90%	0	87.58	981	AF226591.1
Contracaecum rudolphii strain B large subunit ribosomal RNA gene, partial sequence	Contracaecum rudolphii	1133	1133	90%	0	87.53	982	AF226579.1
Contracaecum rudolphii voucher CMP IMBR # 1267-1273.N.2p. V 1-7 large subunit ribosomal RNA gene, partial sequence	Contracaecum rudolphii	1131	1131	88%	0	88.06	960	MT329686.1
Raphidascaris acus isolate FP353 large subunit ribosomal RNA gene, partial sequence	Raphidascaris acus	1109	1109	84%	0	88.42	921	PQ570392.1
Raphidascaris sp. m AC-2021 voucher HWML 112250 large subunit ribosomal RNA gene, partial sequence	Raphidascaris mundeswariensis	1109	1109	92%	0	86.69	1005	MZ611858.1
Raphidascaris acus isolate FP593 large subunit ribosomal RNA gene, partial sequence	Raphidascaris acus	1103	1103	84%	0	88.31	921	PQ570397.1
Hysterothylacium fortalezae isolate 06557 28S ribosomal RNA gene, partial sequence	Hysterothylacium fortalezae	1098	1098	94%	0	86.14	1024	KX098564.1
Contracaecum multipapillatum large subunit ribosomal RNA gene, partial sequence	Contracaecum multipapillatum	1088	1088	90%	0	86.83	982	AF226574.1

Strongyluris calotis genes for 18S rRNA, ITS1, 5.8S rRNA, ITS2, 28S rRNA, partial and complete sequence, specimen_voucher: MPM<JPN>:21151	Strongyluris calotis	1026	1026	100%	0	84.01	4284	LC133188.1
Krefftascaris sharpiloi isolate 2 18S ribosomal RNA gene, partial sequence; internal transcribed spacer 1, 5.8S ribosomal RNA gene, and internal transcribed spacer 2, complete sequence; and 28S ribosomal RNA gene, partial sequence	Krefftascaris sharpiloi	1026	1026	95%	0	84.85	3707	GU245692.1
Krefftascaris parmenteri isolate 4 18S ribosomal RNA gene, partial sequence; internal transcribed spacer 1, 5.8S ribosomal RNA gene, and internal transcribed spacer 2, complete sequence; and 28S ribosomal RNA gene, partial sequence	Krefftascaris parmenteri	1026	1026	95%	0	84.82	2083	GU245688.1

Figure S1

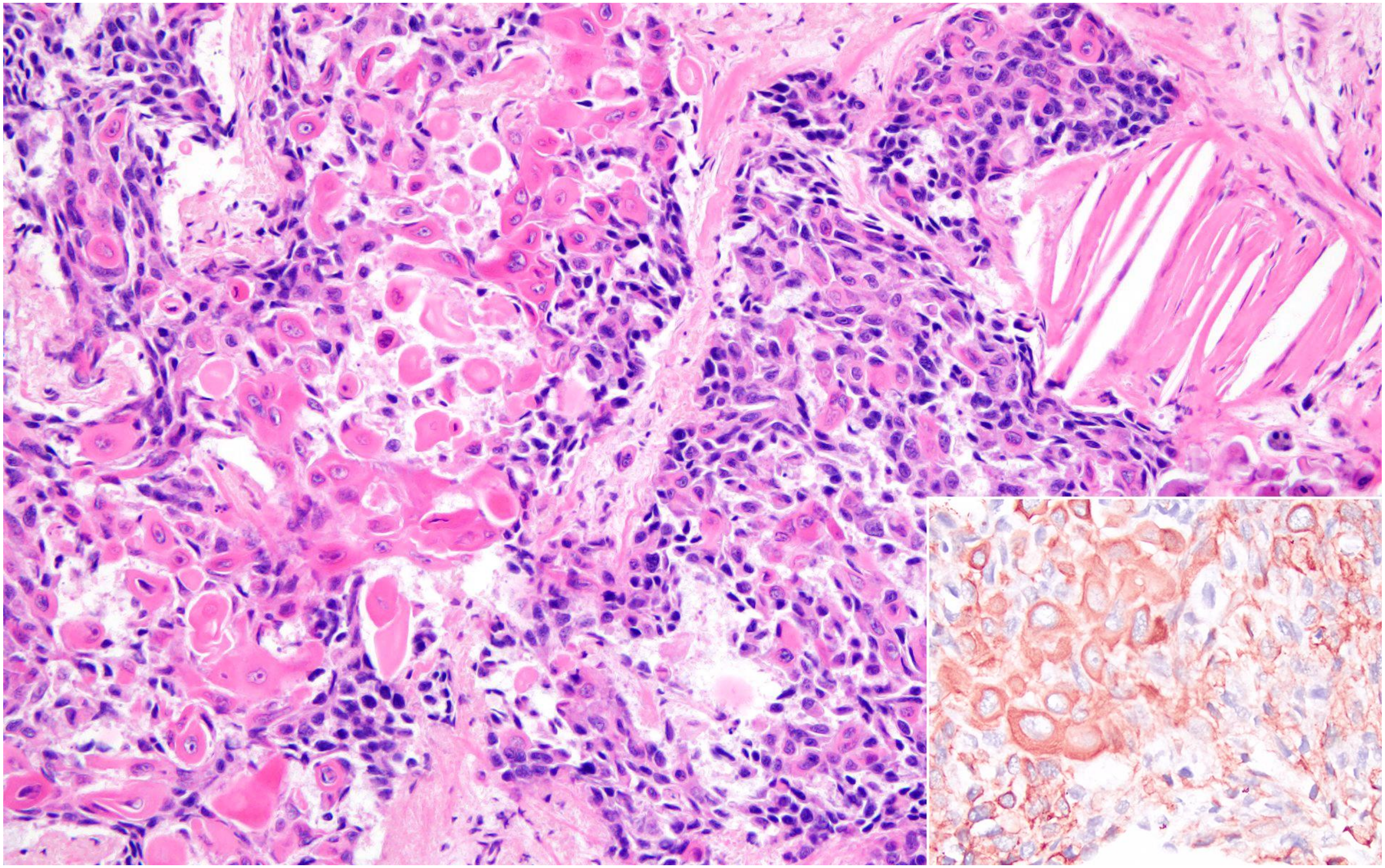

Figure S2

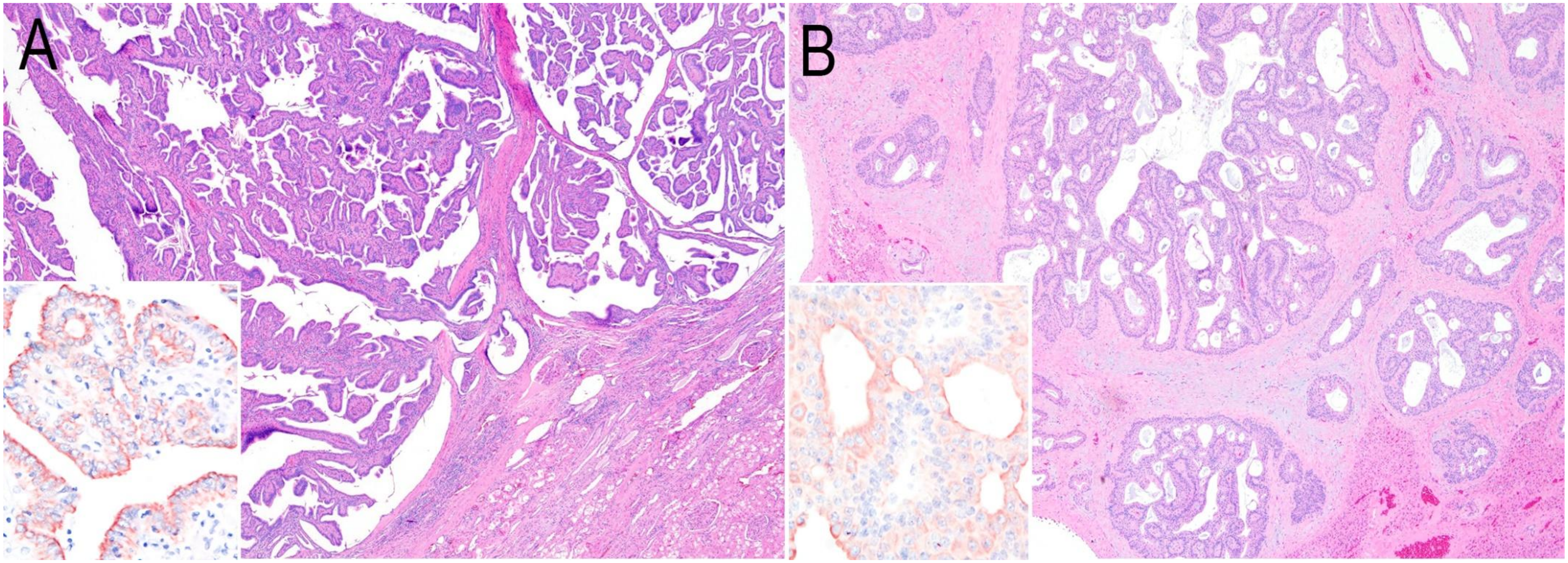

Supplement: Supplementary file 1 [file animals-15-00338-s001.zip › animals-3362534-supplementary.pdf]
